# Glomus Tumor of the Kidney Harboring Malignant Potential

**DOI:** 10.7759/cureus.19479

**Published:** 2021-11-11

**Authors:** Filipos Kapogiannis, Eleni Tsiampa

**Affiliations:** 1 Urology, Hippokration General Hospital, Athens, GRC; 2 Obstetrics and Gynaecology, General Hospital of Helena Venizelou, Athens, GRC

**Keywords:** soft tissue neoplasm, robotic partial nephrectomy, glomus tumor, kidney, malignant

## Abstract

Glomus tumors are rare, benign, vascular neoplasms arising from the glomus body. Although they occasionally develop in any part of the body, they do so more common in the upper extremities, most frequently subungual areas. An extensive review of the literature revealed less than thirty cases of primary renal glomus tumors. We present a unique case of an adult male with an incidentally discovered 2.5 cm right renal mass. Histopathologic and immunohistochemical examination suggested the diagnosis of glomus tumor. Based on the World Health Organization (WHO) classification of soft tissue tumors, the presence of at least two atypical features indicates malignant potential. In this case, deep/visceral location and size greater than 2 cm fulfilled these criteria. Following an uneventful excision and a 12-month follow-up period, the patient remains disease-free with no sign of local or distant metastases.

## Introduction

Glomus tumors account for approximately less than 2% of soft tissue tumors [1]. They most often occur in the subungual region of the fingers as painful nodules in areas abundant in glomus bodies. Visceral organs such as the stomach, liver, lung are infrequently involved [2]. Reports of a glomus tumor in the urogenital system are extremely rare. The glomus is thought to be a modified smooth muscle cell-based on ultrastructural and immunohistochemical characteristics while glomus tumors are mesenchymal neoplasms which are considered benign, solitary lesions. In this study, we report a case of a glomus tumor arising from the kidney which could harbor malignant potential. This case is also the first reported in Greece.

## Case presentation

**Figure 1:**
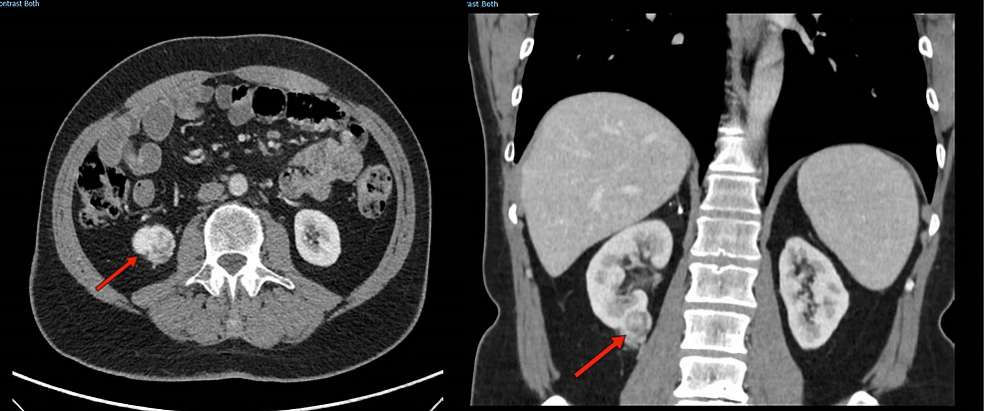
CT scan of the abdomen (axial and coronal plane) demonstrating a 2.5 cm heterogeneously enhancing lesion of the right lower renal pole as designated by the arrow. CT: computed tomography.

A 67-year-old Caucasian male with no past medical history visited our outpatient clinic for urological evaluation. He was referred by his general practitioner, who documented microscopic hematuria after a urine analysis check-up. Abdominal ultrasonography showed a 2.5 cm hyperechoic mass in the right kidney. Physical examination was unremarkable. Subsequent triple-phase computed tomography (CT) showed a heterogeneous, enhancing value, lower pole, exophytic renal mass measuring maximally 2.7 cm (anteroposteriorly) × 2.1 cm (mediolaterally) × 2.6 cm (craniocaudally) (Figure 1).

**Figure 2:**
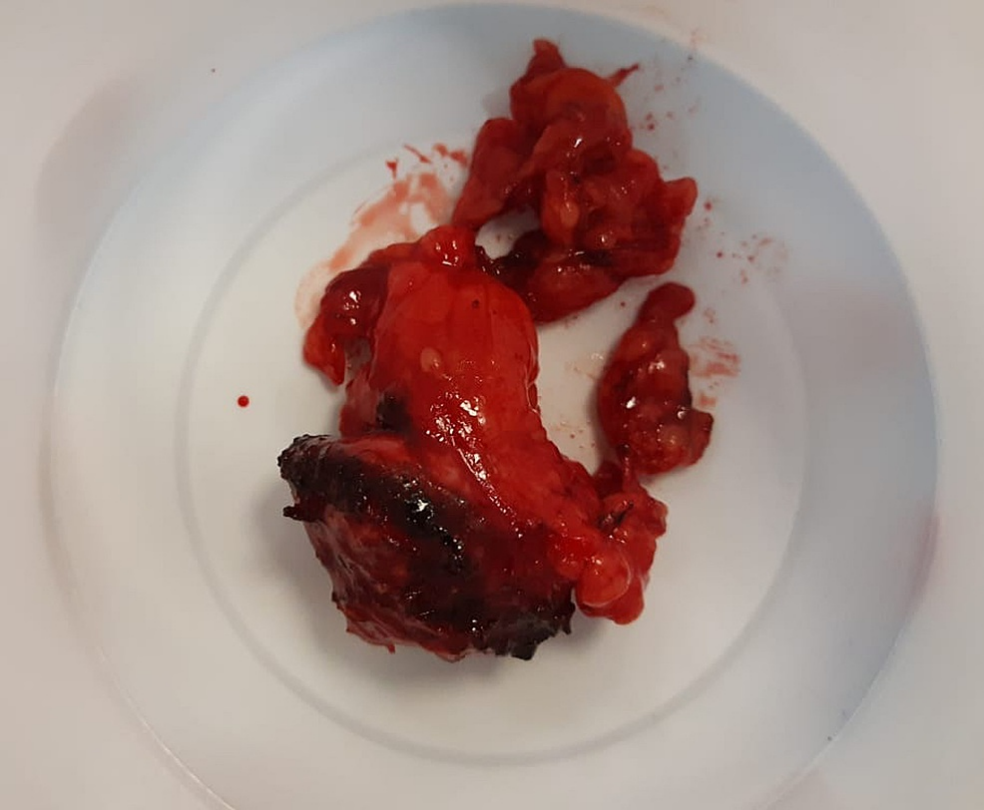
Gross specimen of the renal mass after the partial nephrectomy.

Based on the image findings, it was assumed that the tumor was most likely to be malignant. Therefore we did not measure metanephrines or serum renin levels. After preoperative, routine counseling, the patient refused to undergo a percutaneous, diagnostic renal biopsy and an uneventful, robotic partial nephrectomy was performed. Warm ischemia time was 19 minutes. The gross specimen contained a 25 mm partly exophytic tumor protruding from the capsular surface with a pale and hemorrhagic cut surface (Figure 2).

**Figure 3:**
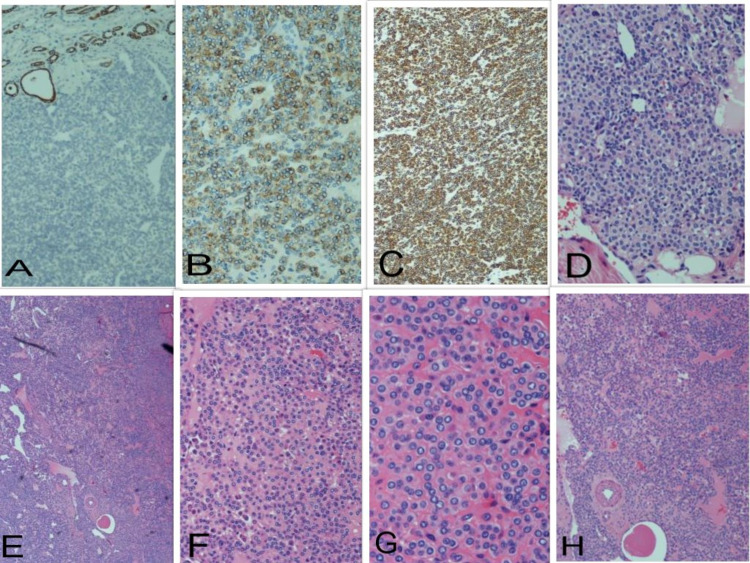
Microscopic evaluation of the excised tumor. H+E-stained sections of the renal mass reveal a monotonous proliferation of cells with round nuclei, small central nucleoli, and a moderate amount of pale eosinophilic cytoplasm with a prominence of cell membranes (D)-(H). Immunohistochemical staining showed the tumor cells to be positive for SMA (C), CD57 (B), and negative for AE1/3 (A). H+E: hematoxylin and eosin. SMA: smooth muscle actin.

Histologic examination showed renal parenchyma containing an unencapsulated tumor, which was generally well-circumscribed but focally more infiltrative, invading the perinephric fat. The lesion comprised of a monotonous proliferation of cells with round nuclei, small central nucleoli, and a moderate amount of pale eosinophilic cytoplasm with a prominence of cell membranes. These were closely associated with variably sized vascular spaces. There were also frequent admixed mast cells. A mild degree of pleomorphism was present but mitotic activity was not identified. Immunohistochemical showed the tumor cells to be positive for smooth muscle actin (SMA), CD57, vimentin and negative for AE1/3, MNF 116, CK7, CK10, PAX8, desmin, CK117, CD31, CD34, CD56, and chromogranin. The proliferation fraction with mindbomb homolog 1 (MIB-1) was approximately 1%. These morphological and immunohistochemical features were consistent with a glomus tumor (Figures 3A-3H).

## Discussion

Soft tissue tumors represent a complex group of lesions that may show a broad range of differentiation. The updated 2020 World Health Organization (WHO) classification, which incorporates detailed clinical, histological, and genetic data include glomus tumors under the lesions in the pericytic or perivascular category [3].

Histologically they are made of up an afferent arteriole, an anastomotic vessel, and a collecting venule. They are modified smooth muscle cells that control the thermoregulatory function of dermal glomus bodies. One should not confuse them with the glomus cells located in the carotid and aortic bodies, which develop paragangliomas.

No specific radiographic feature characteristics of the glomus tumors have been described thus far. Low or high signal intensity may appear on T1- or T2-weighted MRI images, respectively, with vivid enhancement on postcontrast images [4]. To date, no data exist on preoperative needle aspiration or biopsy apart from a case of glomus tumor with multiple organ involvement and aggressive biological behavior at presentation [5].

While glomus tumors are generally considered benign, they can be rarely malignant and may metastasize to bone, brain, liver, lung, small intestine, adjacent lymph nodes, or even recur after surgical excision of the primary lesion. Folpe has proposed a subclassification of atypical and malignant glomus tumors after histologic analysis of 52 cases [6]. The criteria for malignancy were deeply located tumors, size ≥2 cm, atypical mitotic figures, moderate to high nuclear grade, and ≥5 mitotic figures/50 high power field (HPF).

Owing to the paucity of renal glomus tumors, the above-mentioned potential predictors of malignancy cannot be fully corroborated. Firstly, whether the locally infiltrative pattern of growth leads to distant metastases is unknown. Secondly, renal glomus tumors with a size ≥2 cm and a deeper location than the muscularis fascia can follow a benign clinical course [7]. Lastly, due to the short follow-up period of the patients, the implication of possible risk factors with regard to any malignant potential is uncertain and undetermined. In our case, the WHO classification scheme alone qualifies the tumor as a malignant glomus tumor. However, after a 15-month, rigorous follow-up period (including blood workup, chest imaging, and computed tomography urogram), the patient remains disease-free without evidence of a local or distant recurrence.

## Conclusions

Based upon this clinical case, we conclude that the diagnosis of tumor “harboring malignant potential” or of “uncertain malignancy” would be the most appropriate term used, instead of a firm and irreversible definition of a cancerous lesion. Preoperative kidney biopsy could be of clinical utility in diagnosis, treatment planning, and accurate prognostication based upon renal pathology. Long-term follow-up will ultimately reveal the natural history of the mass and provide a precise definition accordingly.
